# Detection of *Toxocara* spp. Eggs in the Soil of Public Places in and Around of Ardabil City, Northwestern Iran

**Published:** 2017

**Authors:** Ali PEZESHKI, Ali HANILOO, Asghar ALEJAFAR, Behnam MOHAMMADI-GHALEHBIN

**Affiliations:** 1. Dept. of Parasitology and Mycology, School of Medicine, Zanjan University of Medical Sciences, Zanjan, Iran; 2. Dept. of Parasitology, Microbiology and Immunology, School of Medicine, Ardabil University of Medical Sciences, Ardabil, Iran

**Keywords:** *Toxocara* spp., Eggs, Soil, Public places, Iran

## Abstract

**Background:**

Human toxocariasis is contained in the list of neglected diseases. The infection occurs after ingestion of embryonated eggs in contaminated soil. The present study was carried out to estimate the extent of soil contamination with *Toxocara* spp. eggs in the public places.

**Methods:**

Soil samples were collected randomly from 41 public places in various parts in and around of Ardabil, Iran, between March 2013 and March 2014. Data were examined by microscopy following sodium nitrate flotation.

**Results:**

Of the 200 collected soil samples, 35 (17.5%) were positive for soil parasites. The eggs of *Toxocara* spp. were found in 14 (7%) soil samples.

**Conclusion:**

This investigation gives baseline knowledge regarding soil contamination with *Toxocara* spp. eggs in Ardabil city and provides information for local control of toxocariasis.

## Introduction

The *Toxocara* spp. larval stage, the etiological agent of human toxocariasis, is a significant medical problem worldwide (1). Infected dogs and cats harboring adult worms can spread more than 50000 embryonated eggs/g feces into human environment every day (2, 3). These eggs need a period of development in the soil to become infective (4) and can be viable in the environment for a long time (5). Therefore, contact with contaminated soil is the most widely identified source of human infection (6, 7). In fact, humans are typically infected by ingestion of embryonated eggs from the soil (8). For this reason, more surveys have been conducted in recent years to evaluate the frequency of *Toxocara* eggs in the soil of public places in various parts of the world (4, 9–11). Several reports of these studies indicated widespread contamination of the soil with the eggs of *Toxocara* spp. contaminated with the dogs and cats feces (7).

Ardabil City has the high density of stray dogs and cats. These animals roam freely in the environment and produce offspring that may contaminate the soil of public areas with *Toxocara* spp. eggs. Additionally, the population of pets is increasing in this city. However, soil contamination with *Toxocara* spp. eggs have been sporadically reported from Iran (12, 13). There is no previous report about soil contamination with *Toxocara* spp. eggs in Ardabil City.

The present research was designed to estimate the extent of soil contamination with *Toxocara* spp. eggs in different public places of Ardabil City, Iran.

## Materials and Methods

This cross-sectional descriptive study was conducted in Ardabil, Iran, the capital of Ardabil province (North West Iran, 38° 14′ N and 48° 17′ E), between Mar 2013 and Mar 2014. The climate of the city is extremely cold, snowy in winter, and temperate in summer with an average annual rainfall of about 304 mm. The mean yearly humidity is approximately 70%.

**Fig. 1:**
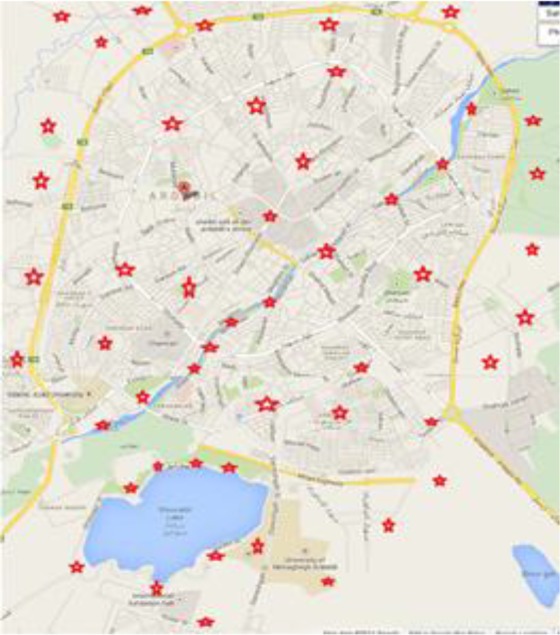
Map showing soil sampling in various parts of public places in Ardabil City, Iran

Overall, 200 soil samples were collected from 41 public places in various parts (North, South, West, East and Central), 40 samples in each parts, of Ardabil City, including sidewalks, public parks, squares, children playgrounds and rubbish dumps by simple random selection (Fig. 1).

Sampling was done in all 4 seasons of the year.

To carry out this, at each sites approximately 150 gram of soil sample was taken with the aid of a small shovel from 8 cm ground depth (14). The samples were placed in sealed and labeled plastic bags and transported to the laboratory. Then, they were dried at room temperature for 2–3 days and passed through a 150 μm mesh sieve.

To recover *Toxocara* spp. eggs, the soil samples were examined by a centrifugal-floatation method with the use of saturated sodium nitrate solution (15). Briefly, 20 gr of the soil sample was placed in a Erlenmeyer’s flask containing 50 ml of 5% NaOH (Merck, Germany) mixed and left for 1h to separate eggs from the soil. The samples were then vortexed for 10min, the suspension was transferred to falcon tube and centrifuged at 1500rpm for 3min. The supernatant was discarded and saturated NaNO3 (Merck, Germany) with specific gravity of 1.30 was added and centrifuged again at 1500rpm for 3min. Finally, the Na-NO3 was added to the tube to form a meniscus and a cover slip was overlaid. After 30 min, the cover slip was transferred onto a microscopic slide.

The preparations were evaluated for presence of *Toxocara* spp. eggs under the light microscope at 100X and 400X magnification. Based on the size and morphology, the parasites were indicated as *Toxocara* spp. eggs or others.

## Results

**Fig. 2:**
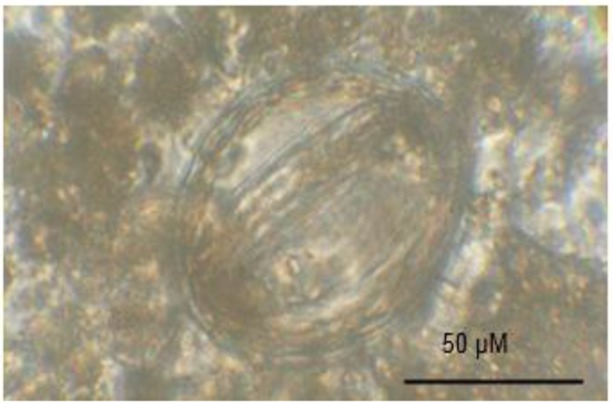
Eggs of *Toxocara* spp. isolated from the soil of Ardabil public places in floatation method

Of 200 soil samples collected in 41 public places, 35 (17.5%) were positive for helminths eggs and nematode larvae (Table 1). Six genera of nematode eggs and larvae (*Toxocara* spp. egg (Fig. 2), *Ascaris* spp. egg, *Trichuris* spp. *egg, Toxoascaris* egg, *oxyur* spp. egg and nematode larvae) and one genera of trematode egg (*Dicrocoelium* spp. egg) were recovered (Table 2). *Toxocara* spp. eggs were the most common helminth egg in the public places of Ardabil City (7%) followed by *Ascaris* spp. eggs (4%) and others (Table 2).

**Table 1:** Contamination rate of soil in the various parts of the Ardabil City, Iran with helminths eggs and nematode larvae

**Zone**	**Number of sites studied**	**Number of positive samples**	**Percent**
Central	8	2	5
North	8	8	20
South	9	19	47.5
East	8	3	7.5
West	8	3	7.5
Total	41	35	17.5

**Table 2:** Type and frequency of helminthes eggs and nematode larvae recovered in various parts of the Ardabil City

**Parasites**	**Number of positive samples**	**Frequency**
*Toxocara* spp. egg	14	7
*Ascaris* spp. egg	8	4
*Trichuris* spp *egg*	5	2.5
Nematode larvae	4	2
*Toxascaris* egg	2	1
Oxyur egg	1	0.5
*Dicrocoelium* spp. egg	1	0.5

The contamination of different parts of Ardabil public places with *Toxocara* spp. eggs are presented in Table 3.

**Table 3:** Contamination rate of soil in the various parts of the Ardabil City, Iran with *Toxocara* spp. Eggs

**Zone**	**Number of sites studied**	**Number of positive samples**	**Percent**
Central	8	1	2.5
North	8	1	2.5
South	9	7	17.5
East	8	5	12.5
West	8	0	0

The burden of *Toxocara* spp. eggs and other detected parasites in current research during the whole year showed that in the spring 6 (12%) out of 50 soil samples were positive for *Toxocara* spp eggs followed by *Ascaris* spp. Egg 1(2%), *Trichuris* spp. egg 1(2%) and others. None of the samples was positive for *Dicrocoelium* spp egg and Nematode larvae.

In the summer *Toxocara* spp. egg was found in 1 (2%) of the soil samples and 1 (2%) of the samples was positive for *Trichuris* spp. egg.

In the autumn 13 out of 50 soil samples were positive for parasitic stages, 4 (8%) out of 200 samples contained *Ascaris* spp. eggs, 4 (8%) of the samples were positive for Nematode larvae, 3 (6%) were positive for *Trichuris* spp. eggs, 1(2%) for *Toxocara* spp. egg and 1 (2%) for *Dicrocoelium* spp. egg.

In the winter 10 out of 50 soil samples contained parasitic stages, *Toxocara* spp. eggs were found in 6 (12%) of the samples and eggs of *Ascaris* spp. were found in 3 (6%) and *Toxascaris* egg was found in 1 (2%) samples (Table 4).

**Table 4:** Parasitic burden of parasites in the soil in public places of Ardabil City in the whole year, Mar 2013 to Mar 2014

**Parasites**	**Spring (%)**	**Summer (%)**	**Autumn (%)**	**Winter (%)**
*Toxocara* spp. egg	6 (12)	1 (2)	1 (2)	6 (12)
*Ascaris* spp. egg	1 (2)	-	4 (8)	3 (6)
*Trichuris* spp. egg	1 (2)	1 (2)	3 (6)	-
Nematode larvae	-	-	4 (8)	-
*Toxascaris* egg	1 (2)	-	-	1 (2)
Oxyur spp. egg	1 (2)	-	-	-
*Dicrocoelium* spp. egg	-	-	1 (2)	-

## Discussion

The ascarids *Toxocara canis* and *Toxocara cati* are common nematodes of dogs and cats, respectively. The soil contamination with *Toxocara* spp. eggs is a significant etiological agent of visceral larva migrans, ocular larva migrans and covert toxocariasis in humans (16). Adult worms of *Toxocara* have been reported in dogs and cats in Iran. Studies have documented the prevalence of 10%–51.6% (17, 18) and 9.4% – 52.7% (8, 19, 20) in dogs and cats, respectively.

This is the first epidemiological survey on frequency of different parasites in soil samples of public places in Ardabil City using centrifugal-floatation method with an emphasis on *Toxocara* spp. eggs.

The presence of *Toxocara spp.* eggs in the soil samples in this survey implied that the stray dogs and cats were infected and defecated in this area. There has been a positive relation between the frequency and concentration of *Toxocara spp.* eggs in the soil and the possibility of human infection (15). Therefore, this alarmingly frequency of *Toxocara* spp. eggs in the soil represents a potential hazard for human. There is no data available regarding the human toxocariasis in Ardabil Province.

The data obtained on the soil contamination rate with the *Toxocara* spp. eggs was as in London/UK (6.3%), Dublin/Ireland (6%), Utrecht/Netherland (7%), Andhra Pradesh/India (6.5%), Pozani/Poland (6.3%), Assam/India (6.12%), Shiraz/Iran (6.3%), different zones/Costa Rica (7%), Urmia/Iran (7.8%) and Qazvin/Iran (5.8%) (9, 21–29).

The rate of contamination in Ardabil City was lower than other studies such as Sao Paulo/Brazil (29%), Kolaczkowwo/Poland (14.5%), Khorram Abad/Iran (22.2%), Erzurum/Turkey (64.28%), Tehran/Iran (10%), Tehran/Iran (38.7%), Bareilly City/India (12.84%), Abadan/Iran (29.2%), Mashhad/Iran (9.2%) and Kermanshah/Iran (18%) (6, 13, 30–37).

The level of soil contamination found in present investigation was higher than reports from Murcia/Spain (1.24%), Melbourne/Australia (1%), Pondicherry/India (2.21%) and Chennai/India (4.75%) (1, 38–40).

The highest frequency of contamination was found in the southern parts of the city. The influence of the location of the area on the environmental contamination is an ongoing controversial issue (41). This fact may be due to the low socioeconomic status of inhabitants and the easy access to dogs and cats in these places.

The frequency of *Toxocara* spp. eggs during summer, and autumn was lower than during spring and winter. Our results are in agreement with previous studies showing the seasonal discrepancy influencing in the prevalence of *Toxocara* spp. eggs in the environment. The differences of contamination rate may be due to culture, geographical parameters, climate condition, seasonal change, soil type, type of population of cat and dog, people’s attitudes toward pets, sample collection, methodology of examination and diagnostic techniques. Thus, the comparison of all such surveys is not reasonable.

## Conclusion

Although, this investigation gives baseline knowledge regarding soil contamination with *Toxocara* spp. eggs and will provide significant information about toxocariasis. Data available about infection in human were limited. Further survey should focus on distinguishing toxocariasis in human in order to develop suitable methods for prevention and control of the disease.
